# The treatment of autoimmune hemolytic anemia with complement inhibitor iptacopan: a case report

**DOI:** 10.3389/fmed.2025.1551042

**Published:** 2025-03-06

**Authors:** Xiaoqing Li, Minran Zhou, Sai Ma, Ran Wang, Chuanli Zhao, Chunyan Chen

**Affiliations:** Department of Hematology, Qilu Hospital of Shandong University, Jinan, China

**Keywords:** iptacopan, autoimmune hemolytic anemia, complement inhibitor, case report, new therapy

## Abstract

Autoimmune hemolytic anemia (AIHA) is a type of hemolytic anemia. In this condition, the body produces anti-red blood cell autoantibodies due to immune dysfunction. This results in accelerated destruction of red blood cells. According to the best temperature for autoantibodies to stick to red blood cells, there are three types: warm antibody type (wAIHA), cold antibody type (cAIHA), and mixed warm-cold antibody type (mAIHA). This article presents a case of acute severe warm antibody autoimmune hemolytic anemia in an elderly male patient. The patient exhibited symptoms including jaundice of the skin, mucous membranes, and urine with a soy sauce color. Laboratory tests were as follows: hemoglobin (HGB) as low as 31 g/L; indirect bilirubin (IBIL) as high as 162 μmol/L; lactate dehydrogenase (LDH) level as high as 1,295 IU/L; reticulocyte percentage (RET%) > 4%; Coombs test positive; conjugated beads protein assay < 0.2 g/L; direct anti-human globulin test positive; indirect anti-human globulin test positive; anti-IgG+++; anti-C3d negative; cold agglutinin test (CAT) negative. After admission, the patient was given a combination of two treatments: methylprednisolone and immunoglobulin. During the treatment, the patient developed a hemolytic crisis. He was immediately given iptacopan and high-dose glucocorticoid shock therapy. After treatment, the patient’s hemolytic-related symptoms improved rapidly. The hemoglobin levels remained within a safe range after stopping the blood transfusion. It is reported as follows.

## Introduction

The first-line treatment for warm antibody autoimmune hemolytic anemia (wAIHA) typically involves glucocorticoids and/or rituximab monotherapy. For patients with severe anemia or those unsuitable for high-dose glucocorticoids, a combination regimen of glucocorticoids and rituximab may be considered, as this approach has demonstrated superior efficacy compared to glucocorticoids alone. In cases of recurrence, intolerance, dependence, or failure of glucocorticoid therapy, rituximab is recommended as the second-line treatment. If the combination of glucocorticoids and rituximab proves ineffective or if the disease relapses within 1 year, third-line treatment options, including splenectomy and cytotoxic immunosuppressive agents, should be explored. Additionally, intravenous immunoglobulin (IVIG) is primarily utilized for AIHA secondary to infections (1, 2).

Iptacopan is a complement factor B inhibitor that targets the amplification loop of the alternative complement pathway. This pathway effectively controls both intravascular hemolysis (IVH) and extravascular hemolysis (EVH) in a comprehensive and potent manner (3). By elevating hemoglobin levels, reducing the need for blood transfusions, and significantly alleviating fatigue (4), iptacopan improves patients’ quality of life. In this case, the patient presented with acute severe hemolysis, and the onset of rituximab was slow. Notably, the complement system, activated via the classical pathway, is implicated in approximately 50% of wAIHA cases (5). In 2018, the first documented case of a C5 inhibitor demonstrated promising efficacy in primary wAIHA (6). Given these findings, iptacopan, a novel complement factor B inhibitor, offers a therapeutic approach by inhibiting the complement cascade at the proximal pathway of complement activation in AIHA. The favorable outcome in this case provides valuable insights for the treatment of similar patients.

## Case presentation

**FIGURE 1:**
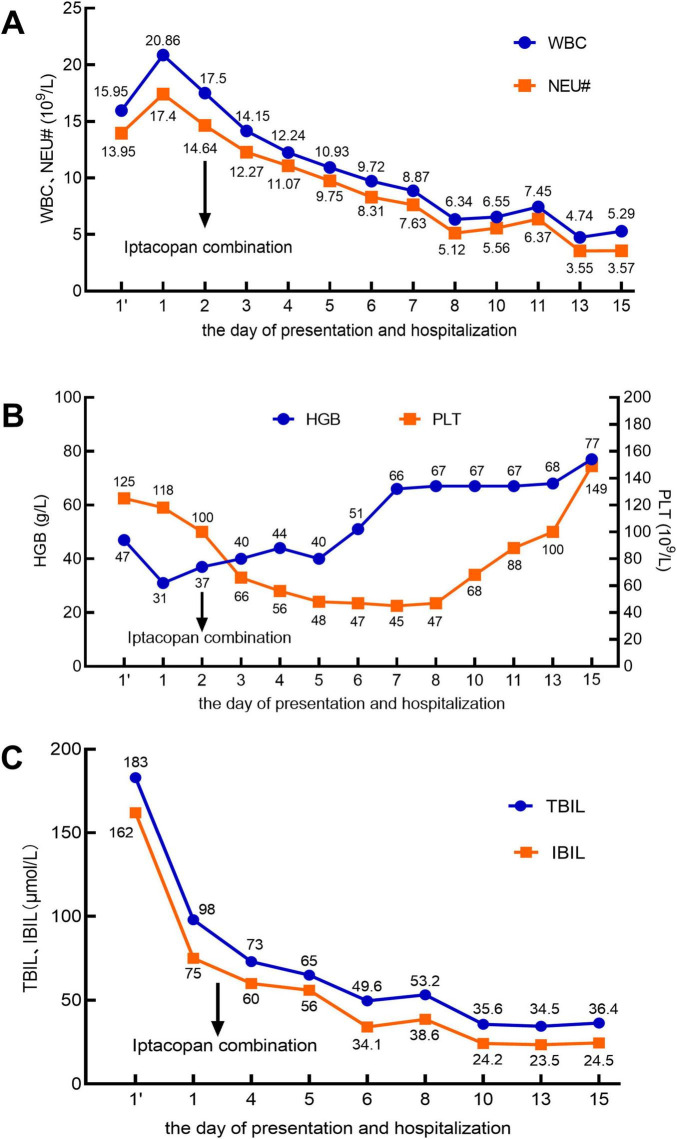
In the X-axis, 1’ (with an apostrophe) denotes the day of presentation, and 1 (without an apostrophe) represents the day of hospitalization. The arrow indicates the initiation of iptacopan treatment on the second day of hospitalization. **(A)** White blood cell (WBC) count (reference range: 3.5–9.5 × 10^9^/L) and neutrophil count (NEU#; reference range: 1.8–6.3 × 10^9^/L). **(B)** Hemoglobin (HGB; reference range: 130–175 g/L) and platelet count (PLT; reference range: 125–350 × 10^9^/L). **(C)** Total bilirubin (TBIL; reference range: 5–21 μmol/L) and indirect bilirubin (IBIL; reference range: 2–15 μmol/L).

**FIGURE 2:**
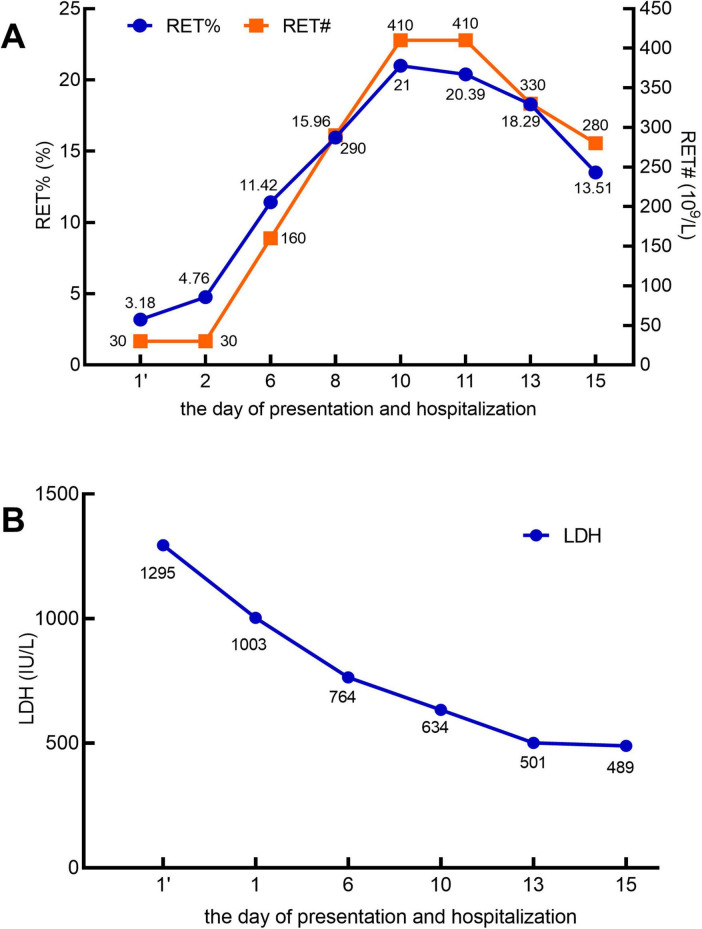
In the X-axis, 1’ (with an apostrophe) denotes the day of presentation, and 1 (without an apostrophe) represents the day of hospitalization. **(A)** Reticulocyte percentage (RET%; reference range: 0.5–2%) and absolute reticulocyte count (RET#; reference range: 20–100 × 10^9^/L). **(B)** Lactate dehydrogenase (LDH; reference range: 120–246 IU/L).

A 60 years-old male patient was admitted to the hospital with a 5 days history of jaundice. Laboratory findings on the day of presentation were as follows: white blood cell (WBC) count, 15.95 × 10^9^/L (Figure 1); neutrophil count (NEU#), 13.95 × 10^9^/L; hemoglobin (HGB) level, 47 g/L; platelet (PLT) count, 125 × 10^9^/L; lactate dehydrogenase (LDH), 1,295 IU/L; absolute reticulocyte count (RET#), 30 × 10^9^/L; and reticulocyte percentage (RET%), 3.18% (Figure 2). Total bilirubin (TBIL) was elevated at 183 μmol/L, with direct bilirubin (DBIL) at 0 μmol/L and indirect bilirubin (IBIL) at 162 μmol/L (Figure 1). Both the direct and indirect anti-human globulin tests were positive. The patient received an emergency transfusion of four units of blood, along with methylprednisolone (80 mg daily) and intravenous immunoglobulin for 2 days in the emergency department.

On the first day of hospitalization, the patient was transferred to the hematology ward, where a series of diagnostic tests were performed. The anti-human globulin test revealed negative results for anti-IgG+++ and anti-C3d. The conjugated bead protein assay value was below 0.2 g/L, and the cold agglutinin test (CAT) was negative. Immunoglobulin levels were as follows: IgG, 17.6 g/L; IgM, 0.205 g/L; kappa light chain, 3.92 g/L; and lambda light chain, 2.24 g/L. Plasma-free hemoglobin was measured at 149 mg/L. Bone marrow cytology and immunophenotyping showed no abnormalities. Tests for cytomegalovirus (CMV), Epstein-Barr virus (EBV), human immunodeficiency virus (HIV), and rheumatologic serology were all negative. Based on these clinical and laboratory findings, the patient was diagnosed with wAIHA.

After 3 days of treatment with methylprednisolone (80 mg daily), the patient’s HGB dropped to 31 g/L, with a RET% of 4.76%. The patient exhibited apathy, severe anemia, jaundice, significant fatigue, dark tea-colored urine, and tachypnea (25 breaths per minute). These clinical and laboratory findings suggested a hemolytic crisis. The acute hemolytic anemia was poorly controlled, indicating an acute severe hemolytic state. Given the slow onset of rituximab, additional therapy was required to rapidly improve the patient’s condition. On the second day of hospitalization, iptacopan (200 mg twice daily) was added to the treatment regimen. On the third day, high-dose methylprednisolone (500 mg daily) was administered for 3 days until the sixth day. Subsequently, the patient continued to receive hormone therapy alongside iptacopan (200 mg twice daily).

From the second to the sixth day of hospitalization, the patient received a total of 10 units of washed red blood cells to correct anemia and 32 units of cryoprecipitate to address coagulation abnormalities. No further transfusions were administered thereafter. Throughout the treatment, the patient received comprehensive supportive care, including intravenous immunoglobulin, hepatoprotective agents, management of jaundice, gastric protectants, cardiotonic medications, anti-infective therapy, and oxygen therapy. The patient’s condition gradually improved, and iptacopan (200 mg twice daily) was continued. On the sixth day of hospitalization, the methylprednisolone dose was reduced from 500 mg to 240 mg daily. Further reductions followed: to 120 mg on day 12 and to 80 mg on day 15.

Throughout this period, no abnormalities were detected on electrocardiogram monitoring. After the sixth day of hospitalization, the patient reported significant alleviation of fatigue, a marked reduction in skin jaundice, and a substantial decrease in the need for blood transfusions. Subsequent laboratory tests revealed that the WBC count had normalized (Figure 1A). The NEU# returned to the normal range by day 8 (Figure 1A). By day 15, HGB levels had increased to 77 g/L (Figure 1B), PLT count had normalized (Figure 1B), IBIL had decreased to 24.5 μmol/L (Figure 1C), RET% had dropped to 13.51% (Figure 2A), RET# had decreased to 280× 10^9^/L (Figure 2A), and LDH levels had declined to 489 IU/L (Figure 2B).

This patient presented with acute severe autoimmune hemolytic anemia, characterized by rapid disease progression. Following treatment with high-dose glucocorticoid pulses combined with iptacopan, both clinical symptoms and laboratory parameters improved significantly, allowing the discontinuation of transfusion therapy. This treatment regimen may provide a valuable reference for the management of similar clinical cases.

## Discussion

In wAIHA, polyclonal immunoglobulins (primarily IgG) bind to RBC antigens. This binding, which is optimal at 37°C, is termed “warm” binding. The pathogenesis of primary wAIHA involves multiple mechanisms, including the role of mononuclear phagocytes in the spleen in erythrocyte destruction (extravascular hemolysis). Additionally, the complement system is implicated in approximately 50% of cases, activated via the classical pathway. IgG triggers complement activation, leading to the production of C3b, which promotes the extravascular hemolysis of opsonized erythrocytes. These opsonized erythrocytes are subsequently captured and phagocytosed by macrophages in the liver. In some cases, C5 cleavage and terminal complement cascade reactions may occur, resulting in the formation of the membrane attack complex (C5b-9, MAC) and intravascular hemolysis (5). In this case, the patient presented with soy sauce-colored urine. Laboratory findings included elevated plasma-free hemoglobin (149 mg/L) and conjugated bead protein levels below 0.2 g/L. However, the CAT was negative, suggesting intravascular hemolysis. These ancillary test results confirmed the occurrence of intravascular hemolysis.

Currently, several novel therapeutic agents are available for the treatment of wAIHA. These include: B-cell targeted therapies: PI3Kδ inhibitor parsaclisib, BTK inhibitor rilzabrutinib, mTOR inhibitor sirolimus, and BAFF-R inhibitor ianalumab. Plasma cell-targeted therapies: Proteasome inhibitor bortezomib. Complement inhibitors: C5 inhibitor eculizumab. Phagocytosis inhibitors: Fostamatinib, sollepineib, and nipocalimab. Among these, eculizumab and fostamatinib have already been approved for clinical use. Parsaclisib, rilzabrutinib, sirolimus, ianalumab, bortezomib, sollepineib, and nipocalimab are currently under investigation in clinical trials (1, 2).

Iptacopan is an oral, potent, and selective inhibitor of complement factor B. It binds specifically to the Bb structural domain of complement factor B, inhibiting its protease activity. Complement factor B is a key protease in the alternative pathway (AP), and the Bb domain constitutes the active component of the AP C3 and C5 convertases. By selectively inhibiting complement factor B, iptacopan blocks C3 activation and the formation of the membrane attack complex (MAC), thereby exerting its therapeutic effects (7). Iptacopan targets complement factor B upstream, inhibiting both complement activation and amplification. This dual action regulates terminal C5-mediated intravascular hemolysis and proximal C3-mediated extravascular hemolysis (3).

In 2024, two phase three trials evaluated the efficacy of iptacopan monotherapy in patients with paroxysmal nocturnal hemoglobinuria (PNH) and hemoglobin levels below 10 g/dL over a 24 weeks period, excluding red blood cell transfusions. The APPLY-PNH trial, a randomized study, focused on patients with persistent anemia despite prior anti-C5 therapy. Its primary endpoint was achieving a hemoglobin level of ≥ 12 g/dL. The APPOINT-PNH trial, a single-arm study, enrolled treatment-naïve patients with lactate dehydrogenase (LDH) levels exceeding 1.5 times the upper limit of normal. Its primary endpoint was a hemoglobin increase of ≥ 2 g/dL from baseline (4). Additionally, the APPLAUSE-LGAN trial, conducted in 2024, demonstrated that patients with IgA nephropathy experienced a 38.3% average reduction in the urine protein-to-creatinine ratio after 9 months of iptacopan treatment (8). These findings highlight the promising efficacy of iptacopan in treating PNH and IgA nephropathy.

A 2018 phase II study of eculizumab in cold agglutinin disease (CAD) included 13 cases. Results showed a reduction in LDH levels from 572 IU/L to 334 IU/L and an increase in HGB levels from 93.5 g/L to 101.5 g/L (9). In the same year, the first reported case of a C5 inhibitor for primary wAIHA involved a combination of hormonal therapy, mycophenolate mofetil (MMF), and 12 injections of eculizumab (ECU). Following the fifth ECU injection, LDH levels decreased significantly from 1,045 IU/L–398 IU/L, with a prolonged remission period of 19 months (6). These studies suggest that complement pathway inhibition is a viable therapeutic strategy for AIHA.

Numerous studies have demonstrated that the most common adverse events associated with iptacopan include headache, abdominal discomfort, and infections (10). Previous clinical trials reported thrombocytopenia in 20% of 10 patients treated with iptacopan, while 90% of patients experienced at least one adverse event, typically mild in severity (10, 11). In the APPLY-PNH trial, thrombocytopenia was observed in 5% of 62 patients, and serious adverse events occurred in 10% of patients. Notably, no patients discontinued treatment due to adverse events (10, 12). However, these trials did not assess whether platelet counts returned to normal levels after treatment. In this case, the patient experienced a transient decrease in platelet count following iptacopan administration, which was attributed to the drug. On the 12th day of iptacopan treatment, the platelet count was near normal, but it returned to normal levels by the 14th day of iptacopan therapy. No further serious adverse events were reported during this period.

The use of iptacopan, a recently approved proximal complement factor B inhibitor for AIHA, has not been previously documented. In this case, during a 15 days treatment period, the patient exhibited significant improvement in hemolytic symptoms, marked alleviation of fatigue, and normalization of laboratory parameters. Additionally, the patient no longer required blood transfusions. These findings suggest that the combination of iptacopan and glucocorticoids holds clinical promise for the treatment of AIHA. However, the available follow-up data for this case are limited. Further validation is necessary to confirm the efficacy of complement factor B inhibitors in AIHA. Additionally, elucidating the mechanisms underlying their therapeutic effects is critical to expanding treatment options, particularly for patients with refractory disease.

## Follow up

Five days after discharge, the patient’s laboratory results were as follows: HGB, 74 g/L; RET%, 8%; TBIL, 42.5 μmol/L; IBIL, 34.8 μmol/L; LDH, 391.2 IU/L; and PLT count within the normal range. Following stabilization, the patient was maintained on low-dose hormone therapy (80 mg daily). However, due to a prolonged history of hormone use, the patient developed complications, including a lung infection and osteoporosis. These were managed with anti-infective therapy and calcium supplementation. One month after discharge, follow-up tests revealed a reticulocyte count of 1.1% and an absolute reticulocyte value of 29 × 10^9^/L. Both TBIL and IBIL levels had normalized, and hemoglobin levels indicated mild anemia. The platelet count remained within the normal range, and the dose of hormone therapy had been reduced.

## Data Availability

The raw data supporting the conclusions of this article will be made available by the authors, without undue reservation.
